# Sequence-based identification of recombination spots using pseudo nucleic acid representation and recursive feature extraction by linear kernel SVM

**DOI:** 10.1186/1471-2105-15-340

**Published:** 2014-11-20

**Authors:** Liqi Li, Sanjiu Yu, Weidong Xiao, Yongsheng Li, Lan Huang, Xiaoqi Zheng, Shiwen Zhou, Hua Yang

**Affiliations:** Department of General Surgery, Xinqiao Hospital, Third Military Medical University, Chongqing, 400037 China; Institute of Cardiovascular Diseases of PLA, Xinqiao Hospital, Third Military Medical University, Chongqing, 400037 China; Institute of Cancer, Xinqiao Hospital, Third Military Medical University, Chongqing, 400037 China; Department of Mathematics, Shanghai Normal University, Shanghai, 200234 China; National Drug Clinical Trial Institution, Xinqiao Hospital, Third Military Medical University, Chongqing, 400037 China

## Abstract

**Background:**

Identification of the recombination hot/cold spots is critical for understanding the mechanism of recombination as well as the genome evolution process. However, experimental identification of recombination spots is both time-consuming and costly. Developing an accurate and automated method for reliably and quickly identifying recombination spots is thus urgently needed.

**Results:**

Here we proposed a novel approach by fusing features from pseudo nucleic acid composition (PseNAC), including NAC, n-tier NAC and pseudo dinucleotide composition (PseDNC). A recursive feature extraction by linear kernel support vector machine (SVM) was then used to rank the integrated feature vectors and extract optimal features. SVM was adopted for identifying recombination spots based on these optimal features. To evaluate the performance of the proposed method, jackknife cross-validation test was employed on a benchmark dataset. The overall accuracy of this approach was 84.09%, which was higher (from 0.37% to 3.79%) than those of state-of-the-art tools.

**Conclusions:**

Comparison results suggested that linear kernel SVM is a useful vehicle for identifying recombination hot/cold spots.

## Background

Meiotic recombination is a vital biological process in diploid organisms, which could be described by two processes: meiosis and recombination. During the former one, the genome is divided into two gametes for sexual reproduction, while diverse gametes combined together to form new genetic variations during the latter. Initiated by double-strand breaks (DSB), recombination provides chances for the natural exchanges of genetic material [1]. By segregating advantageous and deleterious genes, it optimizes genotypes as well as accelerates the evolution of sexual reproductive organisms.

Identification of recombination spots is pivotal in understanding the mechanism of the main driving force in the genome evolution process. Recombination usually occurs in some regions of 1 ~ 2.5 kilobase. In order to find whether they share DNA sequences and structural features, plenty of global mapping studies have been performed to map DSB sites on chromosomes [2, 3]. The genomic regions with relatively high frequencies were known as hotspots, while others with relatively low frequencies were coldspots. Studies showed that most positions of hotspots were intergenic. Meanwhile, positions of hotspots were associated with special chromatin structures, such as GC-rich regions, repeats and consensus DNA motifs and dinucleotides bias. Identifying the recombination hot/cold spots is crucial for understanding the mechanism of recombination as well as the genome evolution process. Since experimental methods are time-consuming and laborious, they may fail to deal with large numbers of genomic sequences. Thus, developing efficient and accurate computational approaches to identify recombination hot/cold spots is required.

The computational approaches for identifying recombination hot/cold-spots consist of the following three components: i) feature extraction for sample representation; ii) optimal feature selection; iii) algorithm selection for classification. Finding proper features to represent the sequences is the first step towards building a novel model. In the past, some features have been used to identify the hotspots. For example, K-mer frequencies of nucleotide sequence contents were used as the features to predict hotspots in IDQD model [2]. However, one of the most important problems in this model, as well as in computational proteomics, is the neglect of global sequence-order effect. In order to keep considerable sequence order information of samples in a discrete model, Chou et al. proposed the concept of pseudo amino acid composition (PseAAC) [4–6], which has been applied to many prediction tasks in computational proteomics [7–10], such as prediction of protein S-nitrosylation sites, protein quaternary structural attributes, protein subcellular locations, membrane protein types, etc. To identify the recombination spots, Chen et al. [1] further proposed the concept of pseudo dinucleotide composition (PseDNC) to represent DNA sequences. Inspired by their model, here we proposed the concept of pseudo nucleic acid composition (PseNAC) of DNA sequence to represent DNA sequences.

Feature selection is another critical step in classification. By decreasing the model’s complexity, the selection of the optimal features can reduce the risk of overfitting and enhance the efficiency. Commonly used feature selection techniques can be attributed into three categories: filter, wrapper and embedded methods [11, 12]. The filter methods, such as Euclidean distance, *T*-test and *X*^2^-statistics, eliminate poorly informative features according to their feature relevance score before inputting any classification algorithm. Wrapper and Embedded methods often provide better results than filter methods because they rank the feature values as subsets as well as interact with the respective classification algorithm. Unlike wrapper methods, which depend on a given but separate classification algorithm, embedded methods perform both tasks, feature selection as well as classifier construction. Thus embedded methods, such as SVM-RFE [13], are computationally less intensive than wrapper methods.

Many different prediction algorithms in computational biology, such as support vector machine (SVM), discriminant algorithm, neural network algorithm, k-nearest neighbor algorithm (k-NN), naive bayes, random forest classifier and increment of diversity (ID), have been developed [14–19]. Among them, SVM was proven to be very powerful in many classification tasks due to its efficiency in analyzing large amounts of samples as well as adaptable to new data [20–22].

In the current work, an SVM-based model was developed to further improve the prediction of recombination spots from pseudo nucleic acid composition (PseNAC) of DNA sequence, including NAC, *n*-tier NAC and PseDNC. Before inputting to an SVM classifier, crucial features were selected by a powerful feature selecting tool, SVM-RFE, for reliable and accurate identification of recombination spots. Employing Jackknife test, our method showed improved prediction performance compared to existing methods.

## Results and discussion

### Parameter selection

Before optimizing the regularization parameter *C* in LIBSVM, we should notice that the dimension of initial feature vector would increase exponentially as the number of the most contiguous residue components increased. For example, the dimension of feature vectors was 4^2^ = 16 for the most two contiguous residue components; while it was 4^10^ = 1,048,576 for the ten most contiguous residue components. However, the higher the number of most contiguous residue components was, the higher rate of redundant information was included in feature vector. Due to the high rate of redundant information and limits in computing power, we finally fixed the maximum number as five for the most contiguous residue components.

**Figure 1:**
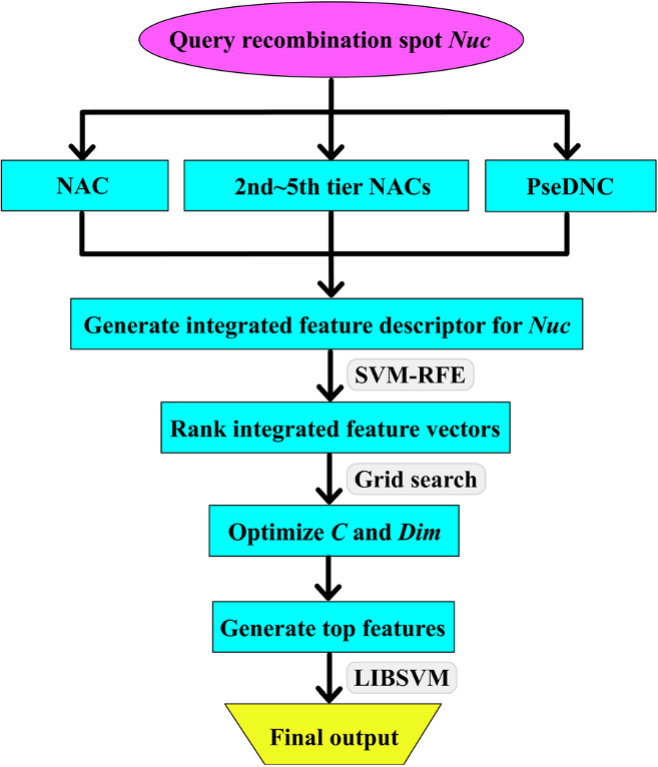
**Comparison of prediction results of different top features.**

The regularization parameter *C* in LIBSVM was determined to compute the prediction accuracy. In this work, we ultilized a grid search approach to select it via computing the best dimension *Dim* of DNA top feature vector. Firstly, we built up an initial feature vector, which was integrated by NAC, *n*-tier NAC and PseDNC of each DNA sequence. Secondly, according to their impact on the SVM model predictions, a ranking list of all the features was returned based on SVM-RFE. According to the ranking list, we computed the prediction accuracies for top *N* features, where *N* = 1,2,3,…200. We found that the accuracy at top106 was the highest for this dataset (Figure 1). Finally, top106 features and the corresponding parameters (*C* = 32 and *Dim* = 106) were chosen as the optimal parameter group to compute the accuracies of our method.

**Figure 2:**
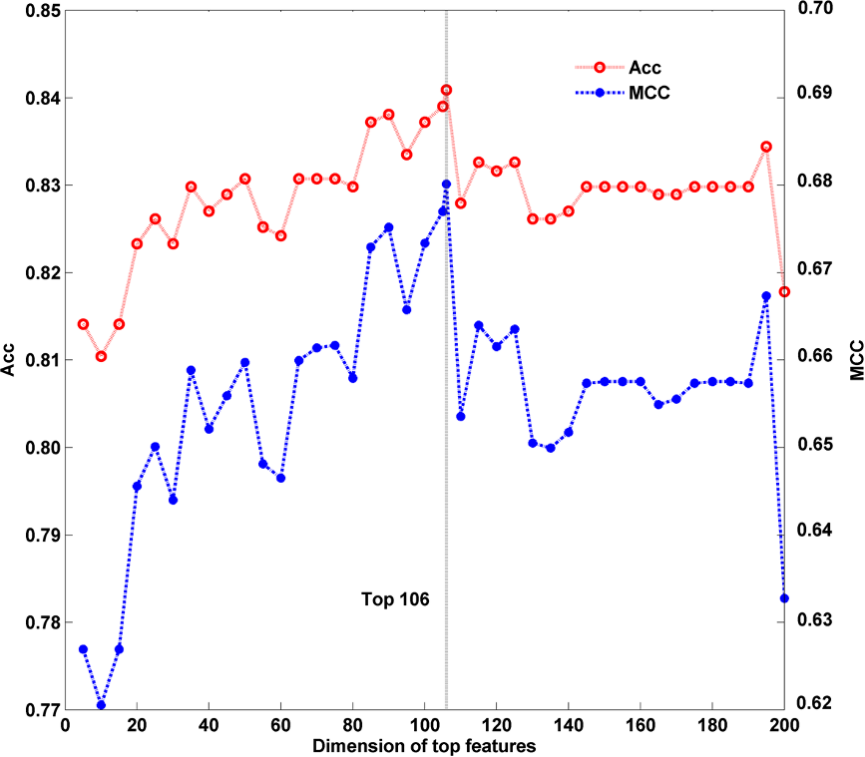
**Top106 features in the benchmark dataset.**

**Figure 3:**
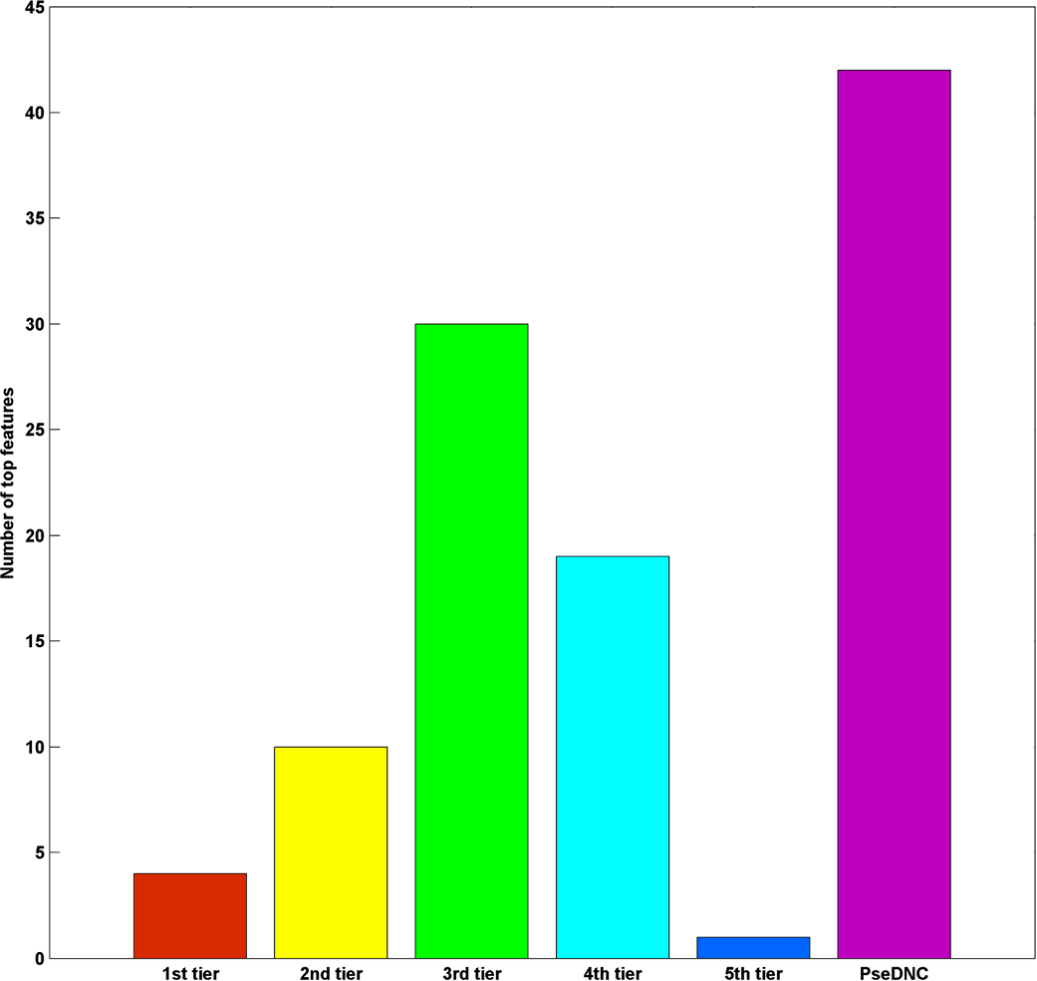
**The overlapped features.**

As shown in Figure 2, 1st ~ 5th tier NACs made up 64 of the top106 features, while PseDNA constitutes the rest. Among the 64 selected NAC features, nearly half of them were 3rd tier NACs, which indicates that the recombination spot identification could be characterized by 1st ~ 5th tier NACs and PseDNA. Of note, top features selected by different datasets could be different, but they had significant overlap. As shown in Figure 3, we randomly divided the benchmark dataset into two parts, i.e., *S*_*1*_ and *S*_*2*_. Then recursive feature extraction method was used for selecting top features based on the two datasets, respectively. After feature selection by SVM-RFE, 26 common NAC features and 20 common PseDNA features were selected in top106 features for *S*_*1*_, *S*_*2*_ and the benchmark dataset.

### Comparison with other methods

**Figure 4:**
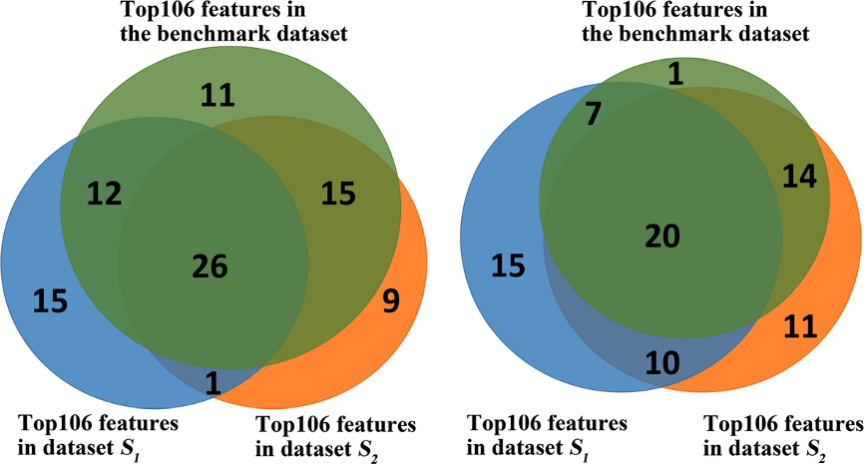
**The ROC curve of the benchmark dataset.**

To assess the prediction performance, we compared our predictor with several previous methods on the same dataset under jackknife test. Our method attained the overall accuracy of 84.09%, which was higher than that with methods [1, 2, 23] listed in Table 1 (from 0.37% to 3.79%). In term of specificity and MCC, our method was also higher than those by other methods. Moreover, we noticed that two other top predictors, iRSpot-TNCPseAAC and iRSpot-PseDNC, also used combined features based on pseudo nucleic acid composition and SVM algorithm, suggesting that the merged features and SVM algorithm were powerful and effective in inferring the recombination hotspots and coldspots. The features in iRSpot-PseDNC only included 16 dinucleotide components and 1 ~ 3 tier correlation factor that reflected the sequence-order correlation between all the most contiguous dinucleotide along a DNA sequence. Obviously, much sequence-order information, e.g., trinucleotide composition and higher tier correlation factors was missed. In order to cover more features, Qiu et al. [23] introduced trinucleotide composition into their predictor, i.e., iRSpot-TNCPseAAC and achieved an overall accuracy of 83.72%. However, integration of more and more features could cause a variety of issues in statistical learning, including the overfitting, dimension disaster, and feature redundancy. Thus an effective feature extraction approach was urgently needed. We compared recursive feature extraction method with another commonly used feature selection method, i.e., F-score. As shown in Table 1, in terms of *Sn*, *Sp*, *Acc* and *MCC*, the former was significantly higher than those by the latter. In this study, recursive feature extraction method could get the key features from high dimension feature vectors more effectively. Accordingly, our predictor performed better than other methods in Table 1 in identifying recombination spots. In addition, to further illustrate the prediction power of our method, a receiver operating characteristic (ROC) curve on the benchmark dataset was implemented (Figure 4). The area under curve (AUC) of our method was 0.703 for the benchmark dataset, which was higher than those by 1–5 tier NACs and PseDNC (AUCs are 0.634 and 0.701, respectively).

**Table 1:** **A comparison of the proposed method with the existing methods**

Predictor	Test method	***Sn***(%)	***Sp***(%)	***Acc***(%)	MCC
The proposed method	Jackknife	76.12	90.69	84.09	0.680
F-score	Jackknife	70.41	88.66	80.39	0.605
iRSpot-TNCPseAAC [35]	Jackknife	87.14	79.59	83.72	0.671
iRSpot-PseDNC [1]	Jackknife	73.06	89.49	82.04	0.638
IDQD [2]	5-fold cross	79.40	81.00	80.30	0.603

## Conclusions

In this study, an SVM-based model was constructed for the identification of recombination hot/cold spots by selecting the optimal features from pseudo nucleic acid composition, *i.e.*, NAC, 2nd ~ 5th tier NACs and PseDNC. The overall accuracy was 84.09% for this benchmark dataset, indicating that this approach was satisfying in identifying recombination sports. It supported the assumption that pseudo nucleic acid composition could better reflect the feature of a DNA sequence through a discrete model, and improved the prediction results for recombination spots identification. Besides, the recursive feature extraction method adopted here was very powerful and effective in getting the optimal features from high dimension feature vectors. Thereore, it improved the final prediction performance as well as accelerated the computing procedure. The good performance of our predictor for identifying recombination spot suggests that our method can be applied as a useful tool in such predicting task. Since user-friendly and publicly accessible web-servers represent the future direction for developing more useful methods, models or predictors, we will make efforts in our future work to provide a web-server for the method presented in this study.

Avowedly, there are still some challenges remaining to be solved in recombination spot identification. Despite the fact that our method suffered from a little high computational complexity for feature ranking, it could effectively catch the key features to improve the identification of recombination spots. In addition, we only focused on the identification of recombination spots, an important step in meiotic recombination. The future attention will be paid in clarifying the relationship between the optimal features selected by this approach and the mechanism of meiotic recombination. As the good performance in identifying recombination spots, we will apply our method to other novel pattern recognition tasks, *e.g.*, prediction of facial features from DNA, DNA methylation level, sparse protein-DNA binding landscapes and small RNA targets, networks and interaction domains.

## Methods

There is no human or animal experiment in this work.

### Benchmark dataset

In this study, the dataset for identifying recombination spots was taken from Liu et al. [2], which contains 490 recombination hotspots and 591 recombination coldspots. It was widely applied as a benchmark dataset for identifying recombination spots [1].

### Feature preparation

Denote *Nuc* as a DNA sequence with *L* nucleic acid residues, i.e.
1

where *R*_*l*_ was the *lth* nucleic acid residue in *Nuc*. Since each nucleotide included a nitrogen-containing nucleobase - either adenine (A), cytosine (C), guanine (G) or thymine (T), we could formulate each DNA sequence *Nuc* by its nucleic acid composition (NAC), i.e.
2

where *F* represented the feature vector of *Nuc. f*(*A*), *f*(*C*), *f*(*G*), and *f*(*T*) were the normalized occurence frequencies of four kinds of nucleobases, respectively. Eq. 2 represented the simpliest features of a DNA sequence. Obviously, all the sequence-order information was lost if only using NAC to represent a DNA sequence. To solve this problem, we adopted dinucleotide composition (DNC) and the feature vector was given by
3

where *f* (*AA*) was the normalized occurence frequency of *AA* in the DNA sequence; *f* (*AC*) was that of *AC*; *f* (*AG*) was that of *AG* and so as *f* (*TT*). In order to capture more local sequence information, the most three, four, five et al. contiguous residue components, i.e., the 3rd, 4th, 5th et al. tier NACs were also incorparated to the PseNAC and similarly we had 4^3^, 4^4^, 4^5^ … features for each DNA sequence. Although the most contiguous local sequence-order information of a DNA sequence was considered, the global sequence-order information was still not reflected. To address this issue, the pseudo dinucleotide composition, i.e., PseDNC was introducted here.

Following the similar procedures in capturizing the global sequence-order information of a protein [24], we extracted global sequence-order information of a DNA sequence formulated by
4

where ∆ represents the coupling mode function as given in Eq.5; *g*_1_ reflects the coupling mode between the most contiguous dinucleotide along a DNA sequence; *g*_2_ is the coupling mode between the second most contiguous dinucleotide; *g*_3_ is the coupling mode between the third most contiguous dinucleotide and so forth. *ω* was the highest rank of the coupling mode along a DNA sequence, and *L*_min_ was the length of *Nuc* with min length in this benchmark dataset. The ∆ function could be formulated by
5

where *J* = 6 was the number of local DNA structural properties as described in ref [25], and *R*_*i*_*R*_*i*+1_ was the 4 × 4 = 6 possible dinucleotides, i.e., AA, AC, AG, AT, …, TT. Table 2 listed the normalized values *V* for the six DNA dinucleotide physical structures, including twist *V*_1_ (*R*_*i*_*R*_*i*+1_), tilt *V*_2_ (*R*_*i*_*R*_*i*+1_), roll *V*_3_ (*R*_*i*_*R*_*i*+1_), shift *V*_4_ (*R*_*i*_*R*_*i*+1_), slide *V*_5_ (*R*_*i*_*R*_*i*+1_), and rise *V*_6_ (*R*_*i*_*R*_*i*+1_). By combining NAC, *n*-tier NAC and PseDNC together, the initial feature vector of a DNA sequence could be represented as
6

where *f* (*A*…*A*) represented the normalized occurrence frequencies of (*A*…*A*), and the length of *A*…*A* was equal to *L*_min_, the minimum length of sequence in the benchmark dataset.

**Table 2:** **The normalized values for the six DNA dinucleotide physical structures**

Dinucleotide	Physical structures					
	V _1_(R _i_R _i+1_)	V _2_(R _i_R _i+1_)	V _3_(R _i_R _i+1_)	V _4_(R _i_R _i+1_)	V _5_(R _i_R _i+1_)	V _6_(R _i_R _i+1_)
AA	0.06	0.50	0.27	1.59	0.11	−0.11
AC	1.50	0.50	0.80	0.13	1.29	1.04
AG	0.78	0.36	0.09	0.68	−0.24	−0.62
AT	1.07	0.22	0.62	−1.02	2.51	1.17
CA	−1.38	−1.36	−0.27	−0.86	−0.62	−1.25
CC	0.06	1.08	0.09	0.56	−0.82	0.24
CG	−1.66	−1.22	−0.44	−0.82	−0.29	−1.39
CT	0.78	0.36	0.09	0.68	−0.24	−0.62
GA	−0.08	0.50	0.27	0.13	−0.39	0.71
GC	−0.08	0.22	1.33	−0.35	0.65	1.59
GG	0.06	1.08	0.09	0.56	−0.82	0.24
GT	1.50	0.50	0.80	0.13	1.29	1.04
TA	−1.23	−2.37	−0.44	−2.24	−1.51	−1.39
TC	−0.08	0.50	0.27	0.13	−0.39	0.71
TG	−1.38	−1.36	−0.27	−0.86	−0.62	−1.25
TT	0.06	0.50	0.27	1.59	0.11	−0.11

### Feature extraction by SVM-RFE

In previous step, NAC, *n*-tier NAC and PseDNC of each DNA sequence were merged as a feature vector. Then, a recursive feature selection approach, SVM-RFE was applied to select a group of important features for reliable identification of recombination spots. Then, through training a linear kernel SVM iteratively, the SVM-RFE algorithm is adopted to get a ranking list of all features by removing only one feature with the lowest influence on the predictions of an SVM model each time [26, 27]. The first item in the ranking list was the most relevant feature in identification of recombination spots, and the last item had the least relevant feature. Finally, the ranking list of the top *K* features was selected to build an SVM model.

### The SVM classifier

SVM is a universal approximator. It is a supervised learning model in analyzing data and recognizing patterns. SVM is attractive to biological sequence analysis due to its ability to handle large input spaces, large dataset and noise. Thus it has been widely used in the bioinformatics applications [28–32]. The basic idea behind SVM is to represent a sample as a point in a high dimensional feature space and then predict it to a category based on the optimal separating hyperplane [33]. In this study, the SVM implementation was based on the package LIBSVM 3.17 [34, 35]. Since the SVM-RFE algorithm was based on a linear kernel SVM, the linear kernel function was applied to obtain the best classification hyperplane. Thus only one free parameter, i.e., the regularization parameter *C* should be optimized. It was determined with an optimal procedure using a grid search method. Finally, the SVM module predicted recombination spots of a DNA sequence using the top features and the optimal value of parameter *C*.

### Assessment of prediction performance

**Figure 5:**
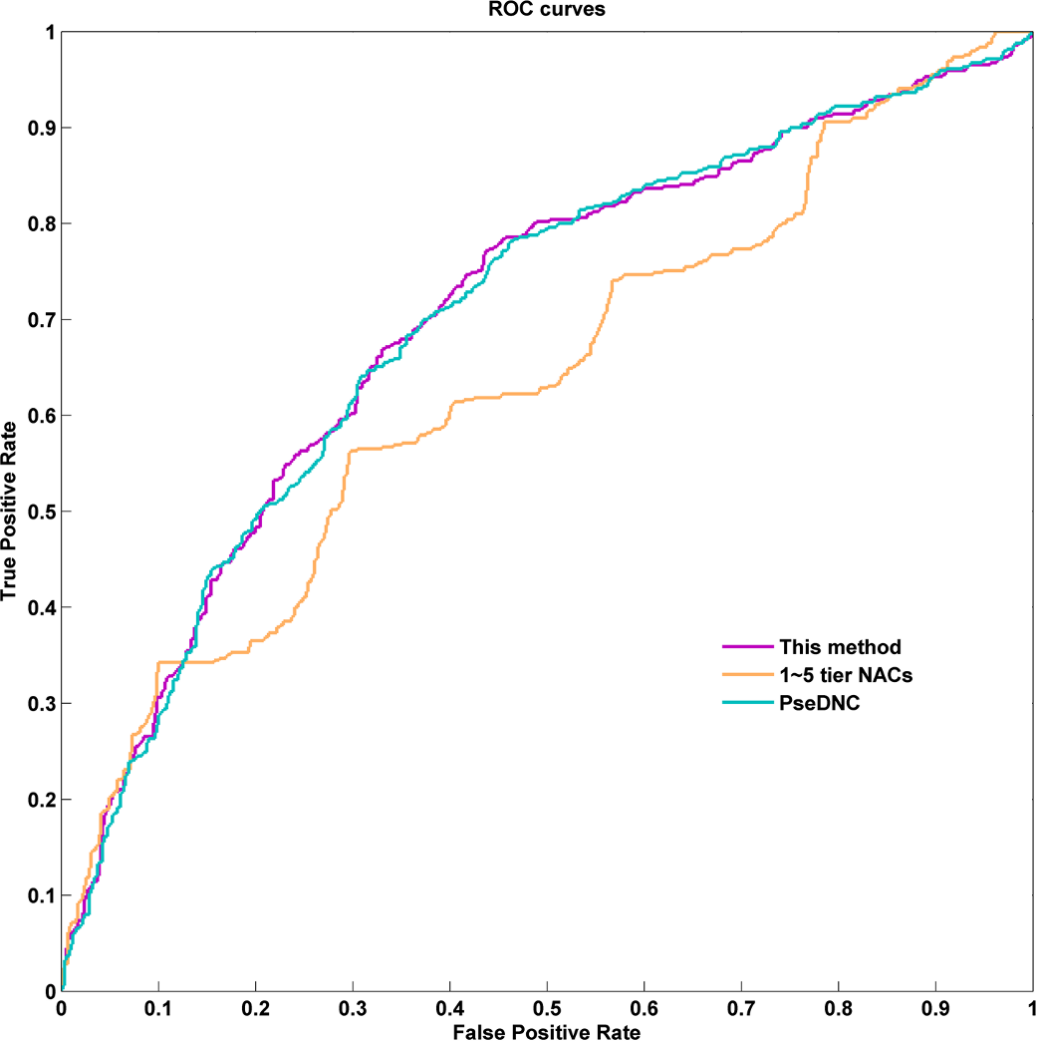
**The pipeline that goes from the query sequence to the final output and all intermediate steps.**

Jackknife test was adopted in this study to evaluate the classification performance of our predictor. In order to make it intuitive and easy for readers to understand, we adopted the formulation proposed recently [5] based on the Chou’s symbol and definition. The sensitivity (*Sn*), specificity (*Sp*), overall accuracy (*Acc*) and Matthew's Correlation Coefficient (MCC) were given by:
78910

where, *N*^*+*^ and *N*^*-*^ represented the numbers of the hotspot and coldspot samples, respectively;  the number of the hotspot samples incorrectly predicted as coldspots while  the number of the coldspots samples incorrectly predicted as hotspot. A flowchart was provided in Figure 5 to illustrate the prediction process of this approach.
